# Association of Coenzyme Q10 Supplementation With Statin-Associated Muscle Symptoms in Long-Term Statin Users

**DOI:** 10.7759/cureus.92705

**Published:** 2025-09-19

**Authors:** Muhammad Zeeshan, Ahmad Raza, Aleena Waseem, Sadia, Muhammad Rehan Mushtaq, Shajee Siddiqui, Hasnat Shabbir, Piash Sarker

**Affiliations:** 1 Medicine, Pakistan Institute of Medical Sciences, Islamabad, PAK; 2 Medicine, Aziz Bhatti Shaheed Teaching Hospital, Gujrat, PAK; 3 General Practice, Fatima Jinnah Medical University, Lahore, PAK; 4 Medicine, DHQ (District Headquarters) Teaching Hospital, Mirpur, PAK; 5 Orthopedics, Luton and Dunstable University Hospital, Luton, GBR; 6 Family Medicine, Amina Healthcare, Sharjah, ARE; 7 Internal Medicine, Jersey General Hospital, St. Helier, JEY

**Keywords:** coenzyme q10, inhibitors, muscles, statins, supplementation

## Abstract

Introduction: Statins are among the most commonly prescribed medications for lowering blood cholesterol levels and are considered safe and effective. However, statin-associated muscle symptoms (SAMS) remain a frequent adverse effect encountered in clinical settings.

Objective: To compare the effectiveness of coenzyme Q10 (CoQ10) with no supplementation in the management of statin-associated muscle symptoms among patients on statin therapy.

Methods: This observational study was conducted in the Department of Medicine at the Pakistan Institute of Medical Sciences (PIMS), Shaheed Zulfiqar Ali Bhutto Medical University (SZABMU), Islamabad, over a period of six months following ethical approval. A total of 106 patients with clinically identified SAMS were included. Group A (n=53) comprised patients who received CoQ10 (50 mg twice daily), while Group B (n=53) included patients who received no supplementation and were managed conservatively.

Results: Patients in Group A who received CoQ10 showed a greater reduction in both VAS and pain interference scores compared to Group B. The differences between the two groups were statistically significant (p = 0.001), suggesting better symptom control in the CoQ10 group.

Conclusion: CoQ10 supplementation (50 mg twice daily) was associated with improved control of statin-associated muscle symptoms in an observational clinical setting. These findings support its potential role as an adjunctive option for patients experiencing muscle-related side effects from statin therapy.

## Introduction

Statins, or HMG-CoA reductase inhibitors, are widely prescribed lipid-lowering agents used in the primary and secondary prevention of atherosclerotic cardiovascular disease. They are well known to be effective in low-density lipoprotein cholesterol (LDL-C) reduction, and they reduce the morbidity and mortality of cardiovascular disease [1]. Nevertheless, long-term usage of statins is often linked with undesirable side effects, of which the most notable effects are statin-associated muscle symptoms (SAMS), which may have a detrimental effect on adherence and the quality of life of the patient. SAMS include a wide variety of muscular complaints and contain, but are not limited to, myalgia, muscle cramps, weakness, and, in extreme cases, rhabdomyolysis [2]. Such symptoms are experienced in about 10-25% of those people taking statins in clinical practice, but less within randomized controlled trials. The pathophysiology is not yet fully clarified, but it is thought to be associated with mitochondrial dysfunction, impaired energy metabolism, and deficiency of the coenzyme Q10 (CoQ10), which is an important part of the mitochondrial electron transport chain [3]. Statins are the most commonly prescribed cholesterol-lowering drugs and are widely used for both primary and secondary prevention of cardiovascular diseases, significantly reducing morbidity and mortality worldwide [4]. Nevertheless, there is a commonly reported negative risk relating to statin therapy, about statin-associated muscle symptoms (SAMS) (myopathy, myalgia, muscle cramps, myositis, or even, in some severe cases, myositis, or even, in some extreme instances, rhabdomyolysis). These adverse effects are major causes of non-adherence and treatment withdrawal, particularly in long-term statin patients [5]. SAMS has an unequal distribution among the populations and methodologies.

Prevalence has been described as up to 9.1% in Europe and up to 17-30% in registry and observational studies in Asia [6]. The incidence of statins-induced myopathy reported in previous studies ranged from 5% to 20% depending on the criteria of diagnosis of myopathy and the type of study [7]. Pakistan has a higher prevalence, including the proportion of SAMS manifestation in patients, which was 51% in a study [8]. The age group 40-50 years was more prone to symptoms, with 47% and 57% prevalence among men and women, respectively. All of them, namely statin, dose, and length, were significantly related to the severity of muscle symptoms (p = 0.05, 0.036, and 0.031, respectively) [9]. The pathophysiological mechanism of SAMS is not fully elucidated yet, but recent evidence confirms that it could be linked to mitochondrial dysfunction, muscle bioenergetics dysregulation, and changes in gene expression [10]. Evidence based on skeletal muscle gene expression in patients with statin-induced myalgia indicates that cellular stress, which is mainly caused by their post-inflammatory repair and sensitivity of end-organs, is a key factor [11]. A likely depletion of one of the most researched mechanisms is the CoQ10, an important mitochondrial cofactor in the electron transport chain and ATP production. In addition to lowering cholesterol, statins also inhibit the mevalonate pathway and thereby decrease the manufacture of isoprenoids, including CoQ10. Mitochondrial dysfunction has been linked to reduced levels of CoQ10 in the plasma and intramuscular environment and could play a role in developing SAMS. Although observational studies and some clinical trials suggest that CoQ10 may alleviate statin-associated muscle symptoms, results from randomized controlled trials (RCTs) have been mixed, with some demonstrating significant benefit while others found no meaningful difference compared to controls [12]. CoQ10 is an endogenous, lipid-soluble antioxidant that offers critical roles in the metabolism of respiration in mitochondria. Granted that it is a common clinical practice, the evidence that proves its effectiveness is ambivalent [13]. As an example, Skarlovnik et al. found a statistically significant benefit of CoQ10 dose of 50 mg bid supplementation on reducing statin-related muscular symptoms and interference scores, implying a clinically significant effect [14]. Conversely, a research study that was conducted by Taylor et al. to assess the impact of CoQ10 on muscle discomfort associated with simvastatin and on the aerobic performance, shows that there is no change in muscular symptoms [7]. 

Objective

To compare the effectiveness of CoQ10 with no supplementation in the management of statin-associated muscle symptoms among patients on statin therapy.

## Materials and methods

This was a prospective observational study conducted at the Department of Medicine, Pakistan Institute of Medical Sciences (PIMS), under the supervision of Shaheed Zulfiqar Ali Bhutto Medical University (SZABMU), Islamabad. Before commencement, ethical approval was obtained from the Institutional Ethical Review Board (ERB), SZABMU, with approval number F. 1-1/2015/ERB/SZABMU/805. All participants were briefed about the purpose and procedures of the study, and informed consent was obtained. The sample size was calculated using the OpenEpi calculator (https://www.openepi.com/SampleSize/SSCohort.htm) at a 5% level of significance and 90% study power. The calculation was based on expected differences in mean pain severity scores between patients who used CoQ10 and those who did not. Based on these parameters, the required sample size was calculated to be 106 patients, with 53 participants allocated to each group. The calculated sample size was 106 patients, with 53 patients in each observational subgroup (CoQ10 users and non-users). A non-probability consecutive sampling technique was used for the recruitment of eligible participants. The study included male and female patients aged between 25 and 60 years who had been using statins for more than six months and reported statin-associated muscular symptoms persisting for at least six months. Only those individuals who were willing to participate and provided written informed consent were enrolled in the study. Patients were excluded if they had alternative identifiable causes of myopathy or were known to have endocrine, renal, hepatic, or vascular diseases. Patients were also excluded if the presence of neurological or orthopedic disorders caused muscular pain (as confirmed by specialist evaluation), prior use of CoQ10 or anticoagulant medications, pregnancy, or an inability or unwillingness to provide informed consent.

Data collection procedure

Patients attending the Department of Medicine at PIMS who fulfilled the inclusion criteria were enrolled. Participants were categorized into two observational groups based on their treatment status. Group A consisted of patients who were prescribed and opted to take CoQ10 supplementation at a dose of 50 mg twice daily, while Group B comprised patients who did not receive supplementation and continued with routine conservative management. Participants were observed under routine clinical care. Those who independently chose or were advised to take CoQ10 (50 mg twice daily) were categorized into the CoQ10 group, while those who did not take any supplementation formed the non-supplemented (control) group. Baseline investigations were performed for all participants, including serum lipid profile, creatine kinase (CK), liver function tests (LFTs), and assessment of pain severity using the Visual Analog Scale (VAS) [15], while pain interference was evaluated using the Subscale of the Brief Pain Inventory (BPI) [16]. After one month of follow-up, the same assessments were repeated. All clinical and laboratory data were recorded in a structured proforma.

Data analysis procedure

Collected data were entered and analyzed using IBM SPSS Statistics for Windows, version 23 (IBM Corp., Armonk, NY, USA). Continuous variables such as age, BMI, lipid levels, CK, and LFTs were summarized as means with standard deviations. Categorical variables like gender were expressed as frequencies and percentages. Pain severity and interference scores before and after one month were compared between the CoQ10 and non-CoQ10 groups using an independent samples t-test. A p-value ≤ 0.05 was considered statistically significant. The findings were presented in tables and interpreted accordingly.

## Results

The results showed a non-significant difference in baseline LDL-C levels between both groups (75.72 ± 15.88 mg/dL in the CoQ10 group vs. 75.04 ± 15.00 mg/dL in the control group, p = 0.821). After one month, LDL-C decreased in the CoQ10 group to 67.70 ± 15.81 mg/dL compared to 71.66 ± 14.71 mg/dL in the control group, though the difference remained statistically non-significant (p = 0.185). High-density lipoprotein cholesterol (HDL-C) levels significantly increased in the CoQ10 group after one month (34.40 ± 3.50 mg/dL) compared to the control group (29.40 ± 3.22 mg/dL), with a p-value of 0.0001. Total cholesterol also showed a significant reduction in the CoQ10 group (92.72 ± 7.28 mg/dL) versus the control group (99.25 ± 7.67 mg/dL) after one month (p = 0.0001), while baseline levels were comparable. Triglyceride levels increased slightly in both groups, with no significant difference observed post-treatment (p = 0.124) (Table 1).

**Table 1:** Changes in lipid profile parameters among statin users with and without coenzyme Q10 (CoQ10) supplementation over one month LDL-C: low-density lipoprotein cholesterol; HDL-C: high-density lipoprotein cholesterol

Parameter (mg/dL)	Group A (CoQ10) baseline (Mean ± SD)	Group A after 1 month (Mean ± SD)	Mean difference Δ (CoQ10)	Group B (Control) baseline (Mean ± SD)	Group B after 1 month (Mean ± SD)	Mean difference Δ (Control)	p-value (Δ between groups)
LDL-C	75.72 ± 15.88	67.70 ± 15.81	–8.02	75.04 ± 15.00	71.66 ± 14.71	–3.38	0.185
HDL-C	25.23 ± 3.48	34.40 ± 3.50	+9.17	25.28 ± 3.28	29.40 ± 3.22	+4.12	0.0001
Total cholesterol	107.00 ± 7.45	92.72 ± 7.28	–14.28	106.42 ± 7.71	99.25 ± 7.67	–7.17	0.0001
Triglycerides	25.15 ± 3.19	35.40 ± 3.53	+10.25	25.09 ± 3.37	34.38 ± 3.23	+9.29	0.124

After one month, alanine aminotransferase (ALT) levels slightly increased in both groups (34.45 ± 2.85 in the CoQ10 group vs. 34.26 ± 3.16 in the control group, p = 0.747), but this change remained non-significant. Similarly, aspartate aminotransferase (AST) levels showed a mild rise post-treatment but were not significantly different between the groups (p = 0.347). Total bilirubin levels showed a slight reduction in the CoQ10 group (0.41 ± 0.09) and a similar trend in the control group (0.39 ± 0.08), again without statistical significance (p = 0.172) (Table 2).

**Table 2:** Changes in liver function tests (LFTs) among statin users with and without coenzyme Q10 (CoQ10) supplementation ALT: alanine aminotransferase; AST: aspartate aminotransferase

LFT parameter	Group A (CoQ10) baseline (Mean ± SD)	Group A after 1 month (Mean ± SD)	Mean difference Δ (CoQ10)	Group B (Control) baseline (Mean ± SD)	Group B after 1 month (Mean ± SD)	Mean difference Δ (Control)	p-value (Δ between groups)
ALT (U/L)	32.17 ± 3.12	34.45 ± 2.85	+2.28	32.32 ± 3.27	34.26 ± 3.16	+1.94	0.747
AST (U/L)	27.09 ± 3.28	31.64 ± 2.30	+4.55	26.79 ± 3.36	32.06 ± 2.23	+5.27	0.347
Total bilirubin (mg/dL)	0.44 ± 0.11	0.41 ± 0.09	–0.03	0.46 ± 0.11	0.39 ± 0.08	–0.07	0.172

At baseline, creatine kinase (CK) levels were similar between the two groups, with Group A (CoQ10) at 39.98 ± 3.24 mg/dL and Group B at 40.40 ± 2.88 mg/dL (p = 0.488), indicating no significant difference. After one month, a significant reduction in CK levels was observed in the CoQ10 group (34.83 ± 3.06 mg/dL), while the group B showed a less pronounced decrease (38.28 ± 2.88 mg/dL), with the difference reaching statistical significance (p = 0.0001) (Table 3).

**Table 3:** Change in creatine kinase (CK) levels among statin users with and without coenzyme Q10 (CoQ10) supplementation

Time point	Groups	Mean ± SD (mg/dL)	p-value
CK at baseline	Group A (CoQ10)	39.98 ± 3.24	0.488
	Group B (Control group)	40.40 ± 2.88	
CK after 1 month	Group A (CoQ10)	34.83 ± 3.06	0.0001
	Group B (Control group)	38.28 ± 2.88	

At baseline, the Visual Analog Scale (VAS) scores for muscle pain were comparable between the two groups, with Group A (CoQ10) scoring 6.47 ± 1.05 and Group B scoring 6.28 ± 1.10 (p = 0.368), indicating no significant difference. After one month of treatment, VAS scores significantly decreased in the CoQ10 group to 1.06 ± 0.84, while the group B showed a more modest reduction to 4.96 ± 0.78. This difference was statistically significant (p = 0.0001), demonstrating that CoQ10 supplementation was associated with a marked improvement in subjective muscle pain (Table 4).

**Table 4:** Visual Analog Scale (VAS) scores for muscle pain in statin users with and without coenzyme Q10 (CoQ10)

Time point	Group A (CoQ10) Mean ± SD	Group B (Control group) Mean ± SD	p-value
VAS at baseline	6.47 ± 1.05	6.28 ± 1.10	0.368
VAS after 1 month	1.06 ± 0.84	4.96 ± 0.78	0.0001

At baseline, Pain Interference Score (PIS) values were similar between Group A (CoQ10) and Group B, with mean scores of 20.42 ± 4.73 and 20.68 ± 4.52, respectively (p = 0.769), indicating no significant difference. After one month, PIS significantly decreased in the CoQ10 group to 7.06 ± 0.79, while the group B showed a lesser improvement with a mean score of 13.55 ± 1.17 (Table 5).

**Table 5:** Pain Interference Scores (PIS) among statin users with and without coenzyme Q10 (CoQ10) supplementation

Time point	Groups	Mean ± SD	p-value
PIS at baseline	Group A (CoQ10)	20.42 ± 4.73	0.769
	Group B	20.68 ± 4.52	
PIS after 1 month	Group A (CoQ10)	7.06 ± 0.79	0.0001
	Group B	13.55 ± 1.17	

## Discussion

Statins are widely prescribed for cardiovascular risk reduction but are often linked to muscle-related complaints, commonly referred to as statin-associated muscle symptoms. Such are muscle cramps, myalgia, and an overall feeling of weak muscles. The diagnosis of SAMS is still not easy, as there can be no confirmatory tests, and thus, the diagnosis is clinical and subjective in nature. Recent data postulate that SAMS may be due to mitochondrial dysfunction, and one of the potential drugs that can be used as an adjunctive therapy is CoQ10. During studies that involved the use of statin medication alone and statin accompanied by CoQ10 supplementation, CoQ10 proved to have positive effects on the recovery of phosphocreatine dynamics, which proves its biological nature in overcoming the muscular problems presented by using statins. This is indicative of the emergence of interest in examining preventative treatments as CoQ10, in the treatment of these symptoms [17]. In the present research, 106 patients were recruited who had used statin drugs chronically, and the period of reporting muscle symptoms was equivalent. The two groups were equal, and one of them got CoQ10 supplements, with the other one being taken as a control group. At baseline, both groups were similar with respect to demographic and clinical characteristics [18].

The findings were a decrease in the total cholesterol and an increase in the level of HDL in the group taking CoQ10; the changes were found to be highly significant. The result was that the LDL levels in the CoQ10 group were lower than those in the placebo group, but there was no statistical significance between the two. The level of triglycerides had a minimal increase, and it was also not statistically significant [19]. The results suggest there is a likelihood that CoQ10 has a lipid-modulating effect, where the short follow-up does not permit conclusions about the long-term lipid outcomes. The results should be reinforced by further observation of lipid profiles [20]. The enzymes in the liver (ALT and AST) were mildly increased in both groups after treatment; however, the result was not significant. It is possible that CoQ10 was not found to contribute to hepatic dysfunction. Likewise, there were no significant differences in the values of total bilirubin, which pointed to the hepatic expertise of the supplement [21]. Creatine kinase is frequently used to determine muscle damage, and in this study, there was a significant decrease in creatinine (Cr), CK values in the CoQ10 tabulated group after one month. It had been previously reported in studies in which patients who were given CoQ10 showed the same results of reduced pain scores and improved measures of quality of life [22]. In another study, CoQ10 supplementation (50 mg twice a day) induced a significant reduction of the PIS scores and a minor positive effect on the cholesterol and liver enzymes concentrations. They had nearly similar findings to ours regarding the dosage, duration of follow up and outcome measures [23].

The results of other trials with the same CoQ10 dosage demonstrated a decrease in the intensity and interference of muscle pain. Some of the studies used placebo controls, but this is not the case in all studies. Nevertheless, our trial contributes to the body of evidence since we used a control study and our research focused on both subjective and objective variables, including pain score and CK value [24,25]. This study also had a number of limitations in spite of positive results. They restricted the follow-up to one month, which might not adequately show the chronic use of CoQ10 as a supplement to the profile of the lipids or muscle symptoms. The study was based on the use of patient-reported outcome measures, which are subjective in nature, and they may be subject to the perception of individual pain or even expectations.

## Conclusions

It is concluded that coenzyme Q10 (CoQ10) supplementation (50 mg twice daily) is associated with improvement in statin-associated muscle symptoms, reflected by lower pain scores and reduced creatine kinase levels, along with favorable changes in HDL and total cholesterol. We have observed all those parameters in our study and suggest studying further the effects of CoQ10 on lipid profile in combination with statins, as well as statin-associated rhabdomyolysis.
